# Evaluation of the Cardiovascular Effects of *Coriandrum sativum* and *Citrus limon* to Treat Arsenic-Induced Endothelial Damage and Hypertension in Rats

**DOI:** 10.3390/life12111842

**Published:** 2022-11-10

**Authors:** Reemal Rana, Malik Hassan Mehmood, Bushra Shaukat, Sidra Shahid, Abdul Malik, Babar Murtaza

**Affiliations:** 1 Department of Pharmacology, Faculty of Pharmaceutical Sciences, Government College University, Faisalabad 38000, Pakistan; 2 Department of Pharmacology, College of Pharmacy, University of Sargodha, Sargodha 40100, Pakistan; 3 Riphah Institute of Pharmaceutical Sciences, Riphah International University, Islamabad 44000, Pakistan

**Keywords:** *Coriandrum sativum*, *Citrus limon*, hypertension, arsenic, toxicity

## Abstract

Based on the vernacular reputation of *Coriandrum sativum* and *Citrus limon* to treat hypertension, this study was designed to explore the cardiovascular effects of *C. sativum* (CS) and *C. limon* (CL) on arsenic-induced hypertension and endothelial damage. Hypertension was induced by arsenic (100 ppm) in drinking water. The crude methanolic extracts of CS and CL were tested for in vivo and in vitro activities using Power Lab. High performance liquid chromatography analysis of CS and CL showed the presence of phenolic compounds. In anesthetized rats, CS (50 mg) and CL (10 mg) showed a marked decrease in blood pressure of 51% and 35%, respectively. Similarly, ascorbic acid (10 mg) also showed a decreased blood pressure (41%). The CS and CL caused complete relaxation (0.003–5 mg/mL) against phenylephrine (1µM) and high K^+^ (80 mM)-induced contraction. The CS and CL, independently and in combination, exhibited marked (*p* < 0.001) attenuation in the blood pressure of the arsenic-induced hypertensive rats when compared with the controls. The beneficial effects of the CS and CL were also observed on lipid peroxidation and eNOS. These data suggest that CS and CL possess significant antihypertensive activity, possibly mediated via endothelium protection, and anti-oxidant effects. Thus, this study provides a rationale for the medicinal use of CS and CL in hypertension and also against arsenic-induced cardiovascular complications.

## 1. Introduction

Cardiovascular diseases (CVDs) are the leading cause of morbidity and mortality worldwide [1]. An estimated 17.9 million people died from CVDs in 2019, constituting 32% of all global deaths. Hypertension is a major risk factor in the pathogenesis of cardiovascular disorders. Different factors have been shown to contribute to the occurrence of hypertension. A positive correlation between arsenic exposure and systolic blood pressure has been reported previously [2]. Arsenic is a naturally occurring metal that is present universally in both organic and inorganic forms. The main exposure to arsenic is through drinking water contaminated with arsenic and irrigation of crops by this water. Arsenic has been linked to human health in causing cardiovascular and respiratory diseases, hepatotoxicity, neurotoxicity, diabetes mellitus and various types of cancer [3]. Sustained exposure to arsenic results in increased systolic blood pressure, impaired endothelial dysfunction due to impaired NO release, increased levels of total cholesterol (TC), triglycerides (TG), low density lipoprotein (LPL), very low density lipoprotein (VLDL), aspartate aminotransferase (AST), alanine transaminase (ALT), bilirubin, urea, creatinine and decreased levels of HDL that may lead to hypertension [4].

Arsenic toxicity is a global health problem affecting many millions of people [5]. Arsenic exposure causes cardiovascular diseases in humans including hypertension. The mechanisms involved in arsenic-induced hypertension are endothelium dysfunction and calcium sensitization disrupting the antioxidant defense mechanism and stimulating beta adrenoreceptors, thus hyper-activating the sympathetic system (enhancing the expression of endothelin-1). Endothelin-1 is involved in the vasoconstriction and fibrosis of the vascular cells leading to the production of ROS [6].

The phytochemical moieties having antioxidant potential might be used against the arsenic-induced hypertension [7]. Plants have a variety of constituents that may have a curative potential. Nearly 47 million people in Pakistan reside in areas exposed to an arsenic concentration greater than the permissible limit [8]. In this study, two indigenous plants, *Coriandrum Sativum* and *Citrus Limon*, were selected as potential agents against arsenic-induced hypertension. *C. sativum* is an herbaceous plant that belongs to the family Apiceae. Due to the presence of multiple bioactive compounds, a wide range of medicinal activities have been attributed to different parts of this herb [9]. *C. sativum* is (CS) is known for its antioxidant, antihyperglycemic [10], anti-inflammatory [11], antihyperlipidemic and diuretic [12] activities. *C. limon* (CL) belongs to the family *Rutaceae.* It is known for its excellent antioxidant, antidiabetic, antihyperlipidemic and antihypertensive properties [13]. Hence, the present study was designed to evaluate the antihypertensive activities of CS and CL individually and in combination in an arsenic-induced hypertension model. This study also explored the possible mechanisms of action through the oxidative stress and nitric oxide pathways.

## 2. Materials and Methods

### 2.1. Chemicals and Reagents

AlCl_3_, Follin-Ciocalteu reagent, gallic acid, quercetin, normal saline, distilled water, formalin 10%, thiobarbaturic acid, sodium chloride, potassium chloride, calcium chloride, sodium bicarbonate, glucose, magnesium sulphate, potassium dihydrogen phosphate, 2, 2-diphenyl-1-picrylhydrazyl (DPPH), acetylcholine, phenylephrine and vitamin C were used in study. All these chemicals and reagents were of analytical grade. Sodium arsenite was purchased from Merck. eNOS ELISA kit was purchased from Elabscience. Methanol used was of HPLC grade.

### 2.2. Plant Collection and Identification

Test drugs *C. sativum* (seeds) and *C. limon* peel were purchased from local botanical store, Faisalabad, Pakistan. Individual samples of plant were positively identified by Dr. Mansoor Hameed in the Botany department of University of Agriculture, Faisalabad under the voucher numbers 1113-2-1b and 113-20-1a.

### 2.3. Preparation of Extract of C. sativum and C. limon

The seeds of *C. sativum* (1 kg) and whole *C. limon* peel (0.5kg) were dried, cleaned and ground in electric grinder. Then, both plants were soaked separately in 80% aqueous methanol at room temperature for 3 days with intermittent shaking. The extracts were filtered through a muslin cloth and then through a filter paper. The procedure of soaking and filtration was repeated thrice. The combined filtrate was then processed in rotary evaporator. Both extracts were soluble in distilled water. The gained extract of CS was thick, sticky semi-solid and of light brownish color [14,15]. The extract of CL was non-sticky, brownish-black in color and of semi-solid consistency [16]. Percentage yields of methanolic extracts of CS and CL were calculated to be 5% and 10%, respectively, by using the formula:

% yield = (actual yield/theoretical yield × 100)(1)

### 2.4. High Performance Liquid Chromatography

The HPLC was carried out to quantify phenolic constituents of *C. sativum* and *C. limon* (Shimadzu, Japan detector: SPD-10 AV, pump LC-10 AT). Shim pack HPLC CLC-ODS C_18_ column of 25 cm × 4.6 mm length and width was used to perform this test. Two types of mobile phase were used named A and B. Mobile phase named as A was acetonitrile, which was run as 15% from 0 to 15 min, as 45% from 15 to 30 min and as 100% from 30 to 45 min. Second mobile phase named as B contained distilled water: acetic acid (96: 4) and its pH was adjusted at 2.27. Ultraviolet visible detector (Model: SPD- 10 AV) was used to detect sample at room temperature at a wavelength 280 nm. To draw a chromatogram, voltage units were plotted along *x*-axis and time in minutes plotted along *y*-axis. Compounds were identified by comparing previously obtained peaks of standards with samples. Compounds were quantified by external standardization method [17].

### 2.5. Determination of Total Flavonoid and Phenolic Content

The plant extract (0.5 mL), 2ml of distilled water and 0.15 mL of 5% NaNO_2_ solution were mixed and incubated for 6 min. Then, 0.15mL of 10% AlCl_3_ solution was added and the mixture was incubated again for 6 min before adding 4% NaOH solution. Volume of the reaction mixture was made up to 5 mL by the addition of methanol and mixed well. Absorbance of the reaction mixture was taken at 510 nm after incubation for 15 min. Total flavonoid contents (TFC) of the extracts were expressed as catechin equivalents from the linear regression curve of catechin.

Follin-Ciocalteu reagent was used to estimate total phenolic content. A total of 200 µL of each of samples was taken in a test tube followed by the addition of 1 mL of Follin-Ciocalteu reagent. After 5 min, sodium carbonate 7.5% *w*/*v* solution was prepared by using distilled water and 0.8 mL was added into the test tube. Solutions were left for 30 min at room temperature for completion of reaction. After 30 min, absorbance of samples was measured at 765 nm by UV spectrophotometer. Activity was performed in triplicate samples and results were obtained by average. Gallic acid was used as a standard. Total phenolic content was calculated from calibration curve of gallic acid expressed as µg gallic acid equivalent (GAE)/mg extract [18].

### 2.6. 2,2-Diphenyl-1-picrylhydrazyl (DPPH) Assay

The antioxidant activity of *C. sativum* and *C. limon* was assessed by DPPH free radical solution. The DPPH free radical assay was performed by following Alhakmani et al., 2013 [19]. The DPPH stock solution (200 µM/L) was prepared in ethanol by dissolving 4 mg DPPH reagent in 50 mL of ethanol and kept in dark. Stock solutions of CS and CL extract (10 mg/mL) were prepared. Stock solution of standard (ascorbic acid) was made of concentration 25 µg/mL. A total of 100 µL of each dilution of test drugs was added in wells of 96-well micro titration plate with help of micropipette and similarly for all standard solutions. Control (100 µL ethanol + 100 µL DPPH) and 200 µL blank (ethanol and distilled water) were used. The change in color from deep violet to light yellow was read at 517nm after 90 min of reaction using an ELISA reader. All assays were performed in triplicate. The percentage inhibition was calculated by using the following formula:

%RSA = 1 − (Abs_control_ − Abs_blank_) (2)

Results were expressed in µg/mL [19].

### 2.7. Animals

Wister albino rats (180–200 g) were purchased from small animal breeding house GCUF, Faisalabad. All animals were placed in cages that were made of polypropylene plastic with a bedding material and maintained under standard laboratory conditions of temperature (25 °C) throughout the experimental period. Animals were given normal feed and tap water ad libitium. Animals were handled according to the guidelines of the Institutional Review Board GCUF, Faisalabad under ref #. GCUF/ERC/2162 (19762).

### 2.8. Experimental Protocol

Rats were divided in seven groups (*n* = 6). First group marked as normal control group, second group as diseased group administered sodium arsenate (100 ppm/kg b.wt) in drinking water, a positive control group as third group given vitamin C, fourth group administered (500 mg/kg b.wt) *C. sativum* methanolic extract, fifth group was administered *C. limon* methanolic extract (100 mg/kg b.wt), sixth group was given a low dose combination of CL and CS (50 + 250 mg/kg b.wt) and seventh group was administered high dose combination of CL and CS (70 + 350 mg/kg b.wt). Animals received sodium arsenate for 12 weeks and plant extracts were administered for last 4 weeks. At the end of experimental study, animals were sacrificed and blood samples were collected for serum collection. Serum was stored at −20 °C until further investigations. Organs (aorta, heart, liver and kidney) were also separated and preserved in 10% formalin for histopathological studies. Organs from other rats were washed with normal saline, weighed (≈100 mg) and homogenized separately by using homogenizer. After homogenization, the homogenate was centrifuged at 2000 rpm for 20 min at 4 °C. The resultant supernatant was collected into separate appendorf tubes to perform lipid peroxidation assay.

### 2.9. Invasive Blood Pressure Measurement

Male rats (200–250 g) were anaesthetized using sodium thiopental injection for invasive blood pressure recordings. A small approximately 1 cm incision was made on mid trachea to expose the trachea, right jugular vein and left carotid artery. Impulsive breathing was maintained through polyethylene tubing of trachea. Tubing was performed to facilitate the administration of drugs, extracts and normal saline. Heparin solution (60 IU/mL) was filled in tube cannulated in carotid artery and on the other end attached with the pressure transducer, which was further connected with the power lab data acquisition system for the blood pressure recording. A total of 0.1 mL of heparin solution was injected to prevent blood clotting. Stable form of animals towards hypotensive and hypertensive responses of Ach and NE was used, successively. A total of 1 µg/kg of acetylcholine in 0.1 mL distilled water was slowly administered by injection followed by push of 0.1 mL saline causing a fall in blood pressure. When the normal blood pressure was regained, norepinephrine was injected slowly at the same amount as acetylcholine followed by 0.1 mL saline causing an increase in blood pressure. Again, after regaining the normal pattern of blood pressure, rats were administered the plant extract doses. After one dose, the other mean arterial blood pressure was permitted to come to its resting level. When plant doses were given, normal saline was flushed after every dose to obtain the true response of next dose. Changes in mean arterial blood pressure were predicted between steady state values before and lower readings after administration [20].

### 2.10. Non-Invasive Blood Pressure (NIBP) Measurement

Blood pressure was measured non-invasively in conscious rats through tail-cuff method by using pulse transducer attached to power lab. Rats were placed in a rat holder with tail placed outside the holder. To keep the rat in stable position, a yellow lamp was positioned above the rat holder. Blood pressure readings were taken 15–20 times. First 10 readings were done to train rat in the process and next 5–10 readings were noted. Systolic and mean blood pressure were noted from computer recordings, whereas diastolic blood pressure was calculated using the formula DBP = (3MBP − SBP)/2 [21].

### 2.11. Blood Collection and Serum Preparation

At the end of study rats were sacrificed using isoflurane. Blood was collected from each rat to obtain serum by centrifugating blood at 7000 rpm and 4 °C for 3 min. Serum was preserved in −20 °C freezer [14].

#### 2.11.1. Serum Analysis of Liver Function (LFT) and Renal Function (RFT)

Renal functions (Urea, Creatinine), LFTs (aspartate aminotransferase, alanine transaminase and bilirubin), were assessed by using commercially available kits (QCA, Tarragona, Spain) with the help of bioanlayzer.

#### 2.11.2. Serum Analysis for Lipid Profile

Lipid profile (total cholesterol, triglycerides, low density lipoprotein, very low density lipoprotein and high density lipoprotein), were assessed using commercially available kits (QCA, Spain) with the help of bioanlayzer.

### 2.12. Malondialdehyde (MDA) Assay

Organs (liver, kidney and heart) were washed with normal saline and homogenized separately in tris-base buffer through homogenizer. The homogenate was centrifuged at 2000 rpm for 20 min at 4 °C. The resultant supernatant was collected into separate tubes for carrying out assay. Reaction mixture was prepared by mixing 25 µL sample, 25 µL sodium dodecyl sulphate (8.1%), 190 µL acetic acid (20%), 190 µL thiobarbaturic acid solution (0.8%) and distilled 75 µL water. This reaction mixture was incubated in water bath for 60 min at 95 °C and left to cool. Then, 625 µL of butanol (15%) solution was added. The mixture was shaken and centrifuged at 4000 rpm for 10 min. Supernatant was separated to measure absorbance at 532 nm using UV-spectrophotometer. Results were expressed in nmol/g wet tissue [22].

### 2.13. eNOS Measurement

eNOS was measured by using ELISA eNOS kit according to manufacturer’s protocol. A total of 200 µL sodium nitrite standard solution was diluted using deionized water and serial concentrations 200, 100, 50, 25 and 0 µmol/L were prepared. Then, in 200 µL serum sample 200 µL reagent #1 and 100 µL reagent #2 were added, mixed fully and left for 15 min at room temperature. The solution was later centrifuged at 3100 g for 10 min. In, 80 µL of chromogenic reagent, 160 µL of supernatant was added, mixed for 2 min and left for 15 min at room temperature followed by the absorbance measurement at 550 nm with micro plate reader [23]. A standard curve was plotted with concentration on *x*-axis and OD on the *y*-axis.

### 2.14. Vessel Tension Measurement

Albino rats were anesthetized using isoflurane in a closed chamber until deep anesthesia was achieved and thoracic aorta was separated. Aorta was cut into 2–3 mm rings that were fixed in a 10 mL organ bath having kreb’s solution at 37 °C. Aorta was continuously supplied with carbogen (O_2_ (95%) and CO_2_ (5%)). Each tissue was allowed to incubate for time period of 60 min after providing a pre-load of 2 g as a baseline tension. Changes in isometric tension were analyzed and recorded using the force transducer, attached with trans-bridge and power lab. Vasorelaxant potential of test materials was tested (0.003 to 10 mg/mL) against phenyl epinephrine (P.E) (1µM) and high K^+^ (80 m M)-induced contractions.

To confirm the integrity of endothelium in diseased and treated rats, aorta was prepared same as mentioned above with slight modifications. Aortic rings were prepared and fixed in organ bath filled with kerb’s solution for the time period of 60 min. Firstly, after completion of equilibration time, the rings were allowed to constrict with phenylephrine (P.E 10^−4^ M) followed by addition of three doses of Ach (10^−7^, 10^−6^, 10^−5^ M) to observe the relaxation. The rings that were constricted more than 50% were progressed to repetition of experiment to ensure that the rings were not eroded due to technical faults [24].

### 2.15. Histopathological Studies

Rats were sacrificed and heart, liver and kidney were stored in 10% formalin solution. Certain sections of organs were stained with haematoxylin and eosin [25] and histopathological observations were performed using Accu-scope 3000-model microscope with an internal ×10 digital camera and further analyzed using Capta Vision software image analyzer.

### 2.16. Statistical Analysis

All results were expressed as mean ± SEM. Results were analyzed using one-way ANOVA followed by Dunnetts hoc test or two-way ANOVA followed by Bonferroni post-hoc test by using Graph pad prism version 5.01, San Diego, CA, USA.

## 3. Results

### 3.1. Yield of Extract

The percentage yield of the methanolic extracts of *C. sativum* and *C. limon* were calculated to be 5% and 10%, respectively.

### 3.2. High Performance Liquid Chromatography Analysis

**Figure 1:**
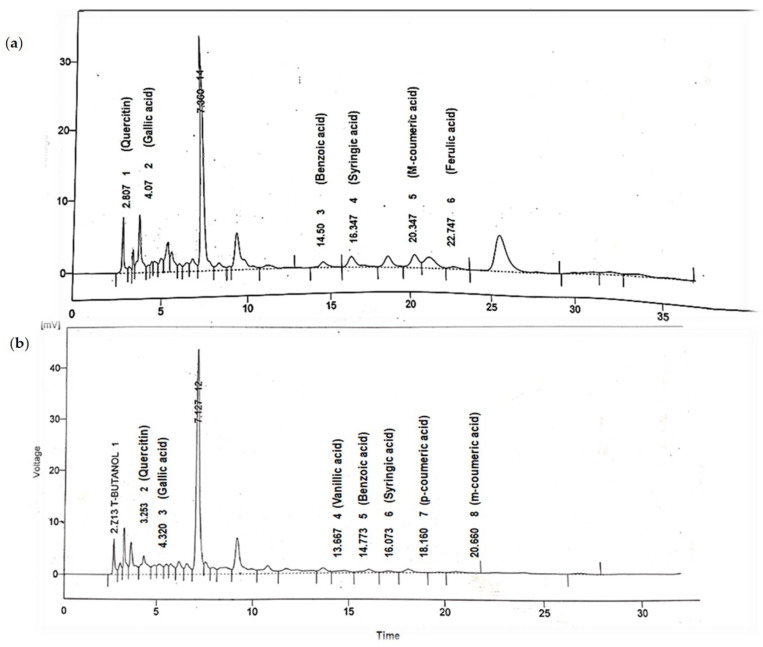
HPLC analysis of *Coriandrum sativum* (**a**) and *Citrus limon* (**b**).

The HPLC chromatograms of *Coriandrum sativum* and *Citrus limon* are shown in Figure 1a,b, respectively. The HPLC analysis of *C. sativum* indicated the presence of quercitin (RT = 2.8 min), gallic acid (RT = 4.60 min), benzoic acid (RT = 14.56 min), syringic acid (RT = 16.34 min), m-coumeric acid (RT = 20.34 min) and ferulic acid (RT = 22.74 min). The HPLC analysis of *C. limon* displayed the presence of quercitin (RT = 3.25 min), gallic acid (RT = 4.32 min), vanillic acid (RT = 13.66 min), benzoic acid (RT = 14.77 min), syringic acid (RT = 16.07 min), p-coumeric acid (RT = 18.16 min) and m- coumeric acid (RT = 20.60 min).

### 3.3. Total Phenolic and Total Flavonoid Content

The total phenolic content in *C. sativum* and *C. limon* was 0.99 ± 0.04 and 0.96 ± 0.11 (µg gallic acid Eq/mg extract), respectively. In contrast, the total flavonoid content in CS and CL was 45.45 ± 3.74 µg/mL and 71.68 ± 3.75 µg/mL, respectively.

### 3.4. DPPH Assay

The free radical scavenging activities of *C. sativum* and *C. limon* were determined by the DPPH radical scavenging activity. The DPPH radical scavenging activity of CS and CL was 73.13 ± 0.59 and 72.00 ± 1.52%, respectively. Similarly, the standard drug ascorbic acid exhibited significant DPPH scavenging activity at 0.25 mg/mL and 55.47% inhibition with an IC_50_ of 14.00 ± 0.14 µg/mL.

### 3.5. Effect of C. sativum, C. limon on Blood Pressure through Invasive Method

**Figure 2:**
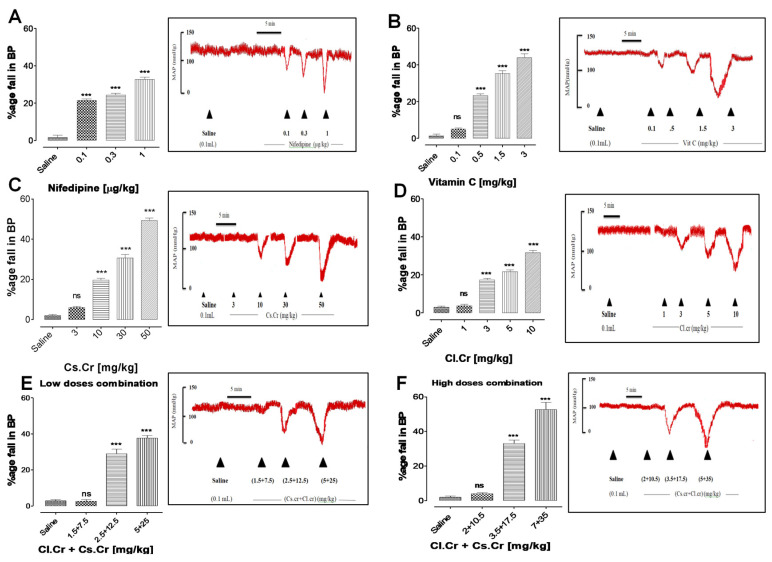
Blood pressure-lowering effect of *C. sativum* and *C. limon* in normotensive anesthetized rats. Effect of nifedipine (**A**), Vitamin C (**B**), *C. sativum* (**C**), *C. limon* (**D**), *C. limon* and *C. sativum* (**E**), *C. limon* and *C. sativum* (**F**) on blood pressure of normotensive rats. Bar chart displays mean ± SEM of 4–5 individual experiments using 3–5 rats. *** *p* < 0.001 when comparison of normal saline (0.1 mL/kg) was done with other groups by carrying out one-way analysis of variance.

When normal saline (0.1ml/kg) was given to the rats, no significant change was observed in blood pressure when normal saline (0.1 mL/kg) was given, while the IV administration of nifedipine and vitamin C, *C. sativum*, *C. limon*, low dose combination and high dose combination caused a percent fall with a maximum effect of 50% in blood pressure. This effect was similar to the effect of nefidipine (positive control). Normal saline was used as the control. The percent fall in blood pressure with nifedipine, vitamin C, CS, CL, low dose combination and high dose combination was compared with the control. The detailed effects on blood pressure are shown in Figure 2.

### 3.6. Effect of C. sativum, C. limon on Blood Pressure through NIBP Method

**Figure 3:**
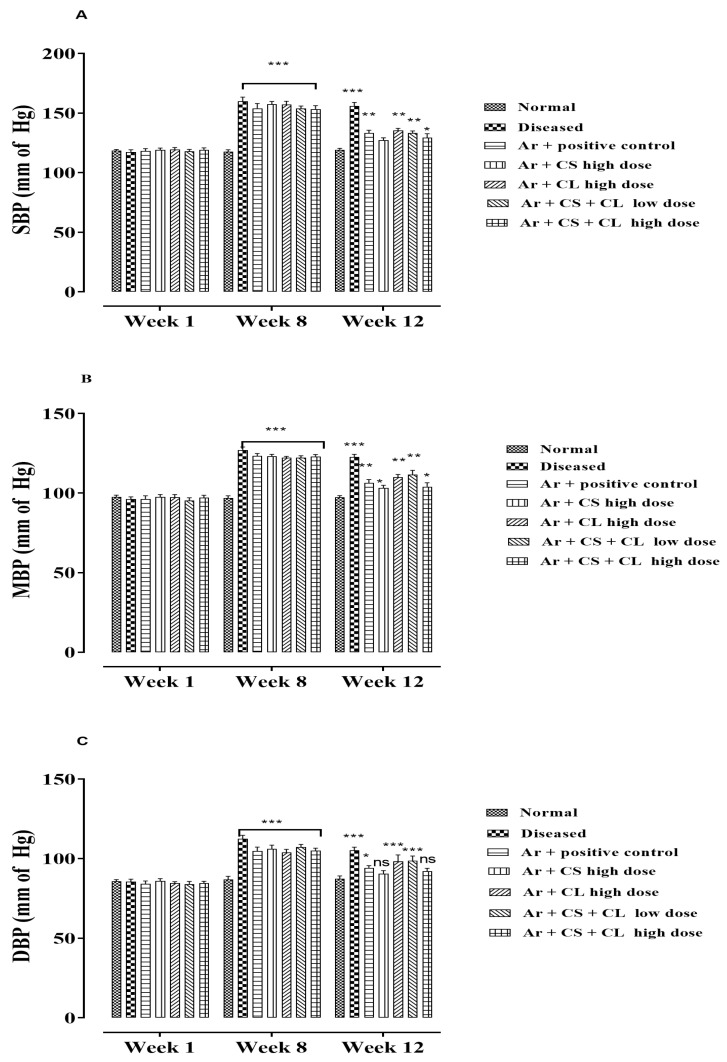
Bar chart illustrates the recorded blood pressure of rats from week 1 to week 12 of study that were fed with arsenic mixed in drinking water. (**A**) systolic blood pressure; (**B**) mean blood pressure; (**C**) diastolic blood pressure. Where normal: normal control. Diseased: arsenic-fed hypertensive control. Ar + CS: arsenic with *C. sativum* (500 mg/kg). Ar + CL: arsenic with *C. limon* (100 mg/kg). Ar + CL + CS (low doses): arsenic with low doses of *C. limon and C. sativum* (50 + 250 mg/kg). Ar + CL + CS (high doses): arsenic with high doses of *C. limon and C. sativum* (70 + 350 mg/kg). Data are represented in mean ± SEM. *n* = 5. ***, **, * are abbreviated as significant effect (*p* < 0.001, 0.01 and 0.05) and ns = non-significant compared to normal group performed by two-way ANOVA followed by Bonferroni post-hoc test.

The oral administration of arsenic in drinking water caused a significant rise (*p* < 0.001) in the systolic, diastolic and mean blood pressures compared to the controls. When tested for chronic administration of *C. sativum*, *C. limon* and their combinations of low doses and high doses in arsenic-induced hypertensive rats, CS alone and in combination (CS and CL) in high doses significantly (P < 0.001) decreased the blood pressure as shown in Figure 3.

### 3.7. Effect of C. sativum, C. limon and Their Combinations on Serum RFTs and LFTs of Arsenic-Induced Hypertensive Rats

**Figure 4:**
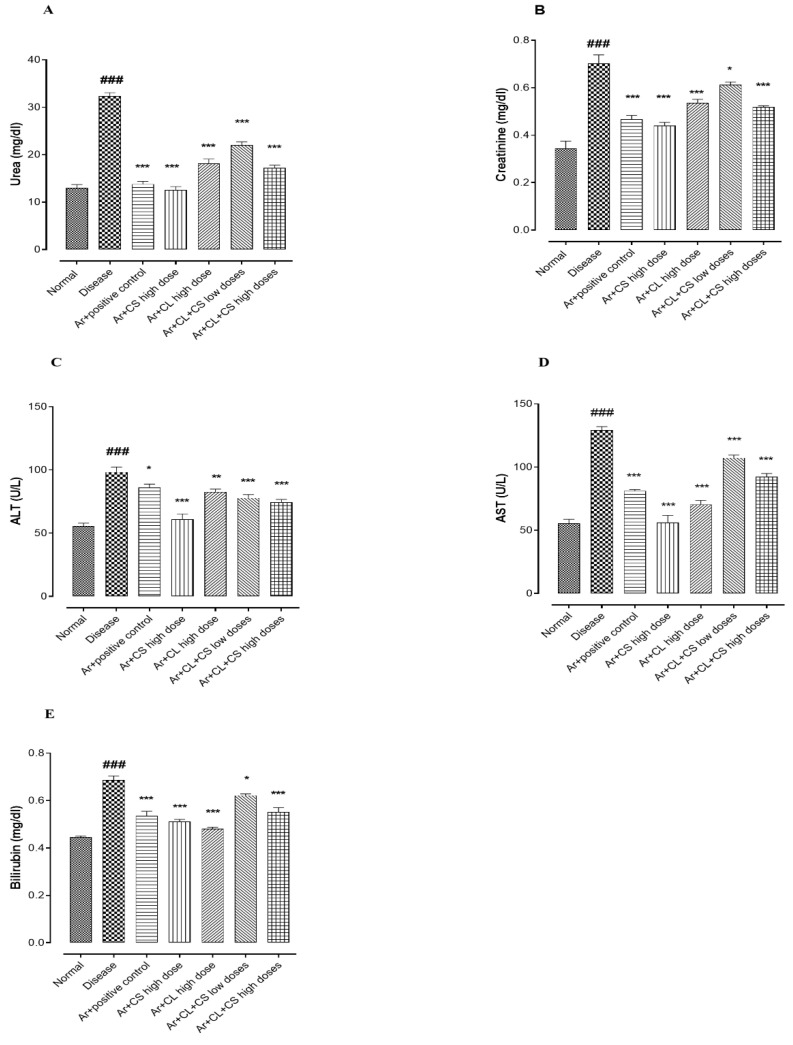
Effect of *C. sativum, C. limon* and their combinations on urea (**A**), creatinine (**B**), ALT (**C**), AST (**D**) and on total bilirubin (**E**) levels. Where normal: normal control. Diseased: arsenic fed hypertensive control. Ar + CS: arsenic with *C. sativum* (500 mg/kg). Ar + CL: arsenic with *C. limon* (100 mg/kg). Ar + CL + CS (low doses): arsenic with low doses of *C. limon and C. sativum* (50 + 250 mg/kg). Ar + CL + CS (high doses): arsenic with high doses of *C. limon and C. sativum* (70 + 350 mg/kg). Data are represented in mean ± SEM; n = 5. ^###^ *p* < 0.001 shows comparison between normal group and diseased group via Student t test. * *p* < 0.05, *** *p* < 0.001 and ** *p* < 0.01 indicate comparison between diseased group and treatment groups by using one-way ANOVA analysis of variance.

The oral administration of arsenic (100 ppm) in drinking water significantly raised the serum RFT and LFT levels (*p* < 0.001) compared to the normal group that was fed with normal drinking water. *C. sativum, C. limon* and their low- and high-dose combinations resulted in significantly ameliorating renal and hepatic parameters including urea, creatinine, ALT, AST and bilirubin in arsenic-induced hypertensive rats as shown in Figure 4.

### 3.8. Effect of C. sativum, C. limon and Their Combinations on Serum Lipid Levels in Arsenic-Induced Hypertensive Rats

Table 1 represents the serum lipid profile of normal, diseased and treated groups of arsenic-induced hypertensive rats. Data are represented as mean ± SEM. The diseased group showed a significant exacerbation of lipid profile (*p* < 0.001) compared to the normotensive control rats. In contrast, the treatment groups showed a significant ameliorative (*p* < 0.001) effect compared to the hypertensive group.

### 3.9. Malondialdehyde Assay

**Figure 5:**
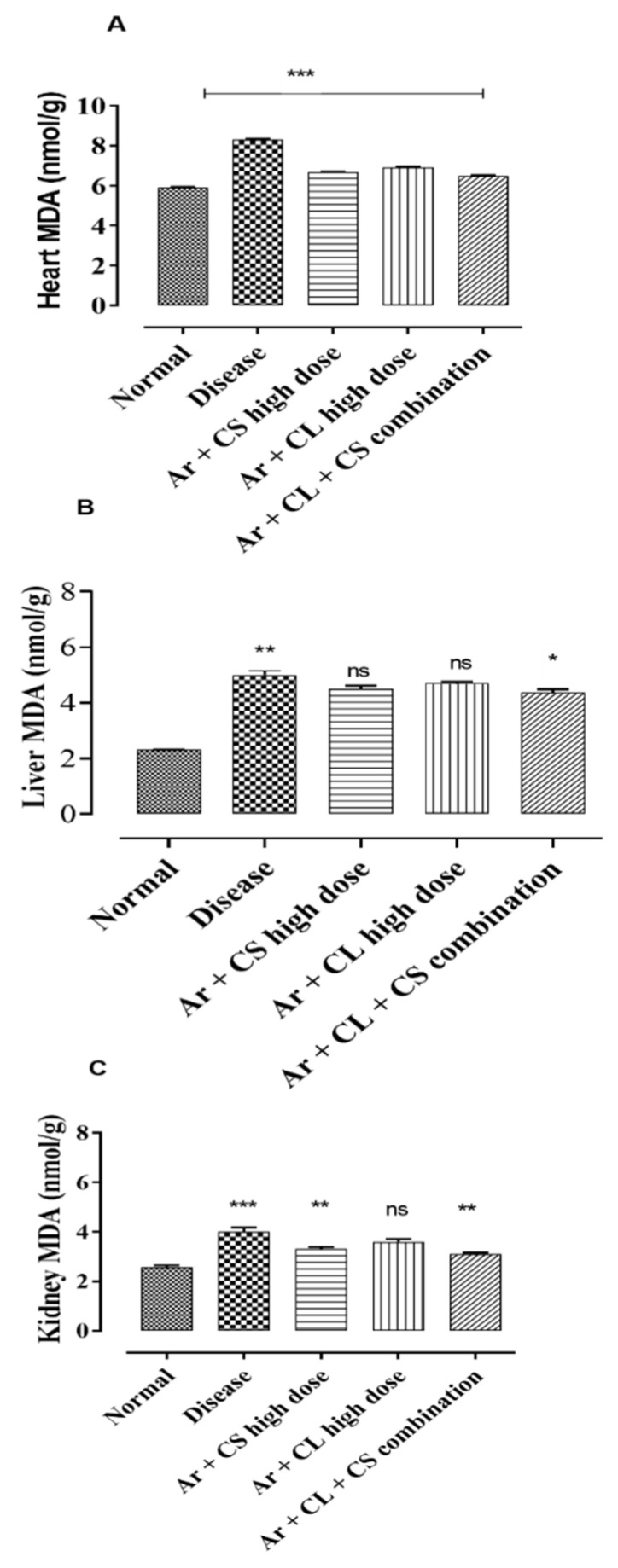
Effect of *C. sativum* and *C. limon* treatment on malondialdehyde (MDA) level in arsenic-induced hypertension. (**A**) MDA level in heart; (**B**) MDA level in liver; (**C**) MDA level in kidney. Where normal: normal control. Diseased: arsenic-fed hypertensive control. Ar + CS: arsenic with *C. sativum* (500 mg/kg). Ar + CL: arsenic with *C. limon* (100 mg/kg). Ar + CL + CS (low doses): arsenic with low doses of *C. limon and C. sativum* (50 + 250 mg/kg). Ar + CL + CS (high doses): arsenic with high doses of *C. limon and C. sativum* (70 + 350 mg/kg). Data are represented as mean ± SEM; n = 5. *** *p* < 0.001, ** *p* < 0.01 and * *p* < 0.05 indicate comparison between diseased and treatment groups by using one-way ANOVA analysis of variance. ns: non-significant difference.

Arsenic administration for twelve weeks resulted in significant gains in MDA levels in the kidney, heart and liver compared to the control rats (Figure 5). The chronic administration of *C. sativum* and *C. limon* alone and in combination resulted in a significant decrease in MDA levels in the heart, liver and kidney tissues as compared with the arsenic-treated rats only (*p* < 0.001).

### 3.10. Effect of C. sativum and C. limon on Serum NO Levels of Arsenic-Fed Rats

**Figure 6:**
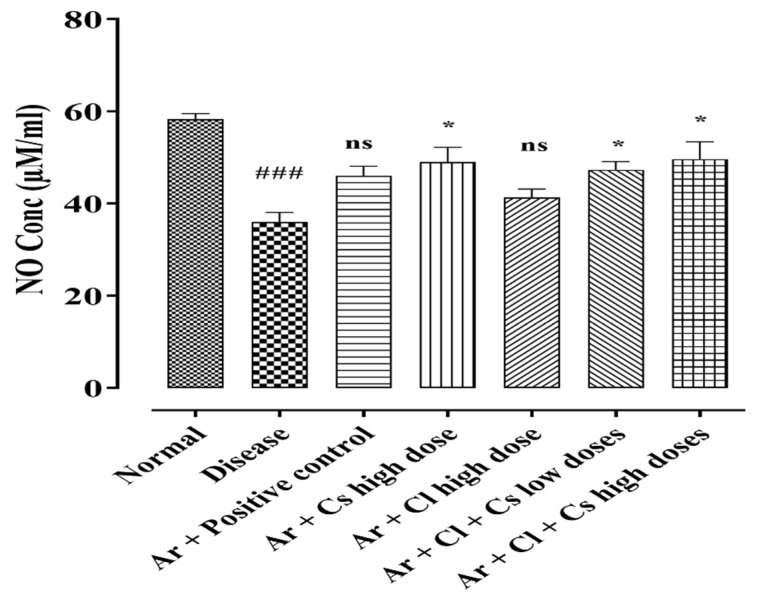
Bar diagram presents the serum NO levels in normal, diseased and treated groups of arsenic-fed rats. Where normal: normal control. Diseased: arsenic fed hypertensive control. Ar + CS: arsenic with *C. sativum* (500 mg/kg). Ar + CL: arsenic with *C. limon* (100 mg/kg). Ar + CL + CS (low doses): arsenic with low doses of *C. limon and C. sativum* (50 + 250 mg/kg). Ar + CL + CS (high doses): arsenic with high doses of *C. limon and C. sativum* (70 + 350 mg/kg). Data are represented in mean ± SEM. ^###^ *p* < 0.001 shows comparison between normal group and diseased group via Student *t* test. * *p* < 0.05 indicates comparison between diseased group and treatment groups by using one-way ANOVA analysis of variance, ns: non-significant difference.

The chronic administration of arsenic resulted in a significant decrease in serum NO levels in the diseased rats (36.00 ± 2.08 µM/mL) compared to the control group (58.33 ± 1.20 µM/mL). *C. sativum* (500 mg/kg), *C. limon* (100 mg/kg) and their low- (50 + 250 mg/kg) and high-dose combinations (70 + 350 mg/kg) were tested for their effectiveness in restoring NO levels in arsenic-induced hypertensive rats. The CS in high dose alone and CL + CS high-dose combination exhibited significant restoration of the NO levels (49.00 ± 3.21 µM/mL and 49.67 ± 3.71 µM/mL) as compared to the diseased rats as shown in Figure 6.

### 3.11. Vessel Tension Measurement

**Figure 7:**
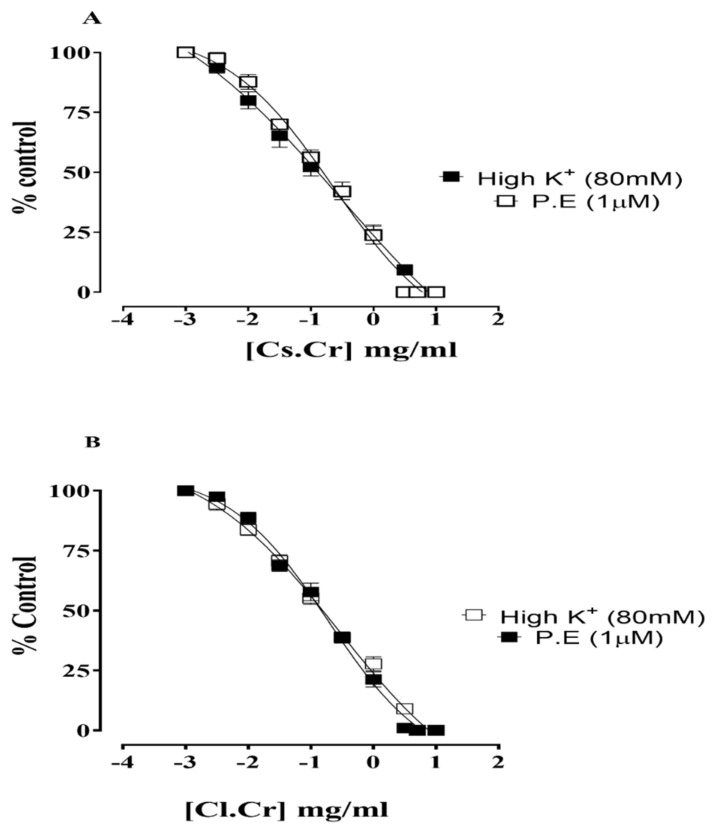
Vasorelaxant activity of *C. sativum* and *C. limon*. % Control relaxation after administering *C. sativum* against high K^+^ and P.E-induced contraction, (**A**). % Control relaxation after administering *C. limon* against high K^+^ and P.E-induced contraction, (**B**).

*C. sativum* and *C. limon* were assessed for their vasorelaxant activities against high K^+^ and P.E-induced contractions at a concentration of 0.003–5 mg/mL with respective log doses of 2.5 to 0.7 mg/mL that exhibited complete relaxation of the pre-contracted vessel. *C. sativum* and CL displayed EC_50_ values with 95% CI intervals of 0.43, 0.27 mg/mL (0.04–2.15, 0.12–0.59) in response to high K-induced contraction. In response to P.E-induced contraction, CS displayed an EC_50_ value with 95% CI intervals of 0.22, 0.18 mg/mL (0.13–0.86, 0.12–0.59; Figure 7).

### 3.12. Histopathological Examination of Heart, Aorta, Kidney and Liver Tissues

The H and E stained pictures of the heart, aorta, kidney and liver are presented in Supplementary File (Figures S1–S4). In the normal heart tissue, intact branching, no cell infiltration and no necrosis were observed. In contrast, in the diseased group branching was not intact, while cell infiltration and necrosis could be seen. The treated groups showed less cell infiltration and decreased necrotic tissue and restored normal branching (Figure S1). As for the aorta, the tissues of the normal animal group exhibited a clear borderline (tunica intima and tunica media), with no mononuclear cell infiltration, and normal collagen and connective tissue, whereas in diseased group, unclear borderline, mononuclear cell infiltration and disorientation of smooth muscles were observed. The treated groups had improved disorientation of smooth muscles and less mononuclear cell infiltration (Figure S2). Normal kidney tissue displayed no cell infiltration and no necrosis. In the diseased group, inflammatory cell infiltration was observed along with fat deposition and damaged tubular structures. The treated groups exhibited less inflammatory cell infiltration, decreased damage in the tubular structure, less fat deposition and improved normal glomerulus (Figure S3). In the case of the liver, the tissue samples from the control rats had intact hepatocytes, normal portal veins, normal portal triads and sinusoids and no necrosis. In contrast, in the case of the diseased group, hepatocyte vacuolization, inflamed portal veins and congested sinusoids were observed. Interestingly, lesser hepatocyte vacuolization, portal vein inflammation and less congestion of sinusoids were observed in the case of the treated groups (Figure S4).

## 4. Discussion

Humans are exposed to arsenic primarily through drinking water and the environment. Arsenic is responsible for various deleterious effects, such as cardiovascular diseases, respiratory diseases, skin cancer, nervous system alterations and intra-uterine disorders [26]. Arsenic contamination in drinking water has been reported in many countries including Pakistan, China, Bengal, India, Nepal, Chile, Mexico, Bangladesh and Argentina, and has detrimental effects on human health. Nearly 47 million people in Pakistan reside in areas exposed to an arsenic concentration greater than the permissible limit [27]. *C. sativum* (CS) is an herbaceous plant belonging to the family *Apiceae*, and *C. limon* (CL) belongs to the family *Rutaceae*. The present study demonstrates the curative potential of CS seeds and CL peel in combating oxidative stress in animals caused by arsenic exposure. The administration to animals with arsenic and plant extracts ameloriated the increase in blood pressure observed in the hypertensive control animals. The doses of CS seed and CL peel used in the current study were 500 and 100 mg/kg, respectively. The low-dose combinations of CS and CL were 250 and 50 mg/kg, whereas in high-dose combination 350 and 70 mg/kg of CS and CL were used. The treatment of animals with arsenic resulted in a significant increase in blood pressure in the hypertensive control animals. The dose of sodium arsenate used was 100 ppm/kg, as reported previously [23].

In this animal study, sodium arsenate induced oxidative stress in animals as indicated by the lipid peroxidation assay. The oxidative stress is produced due to the formation of reactive oxygen species that in turn decrease the anti-oxidant levels. Arsenic activates the NOX_2_ enzyme, a specific NADPH oxidase, and induces the production of ROS. Increased ROS disturbs the normal function of cells and disrupts cellular structures. This, in turn, down-regulates the nitric oxide synthase enzyme and leads to the accumulation of oxidants [28].

Arsenic administration for twelve weeks increased the blood pressure of the animals. Arsenic increases blood pressure through several mechanisms, such as oxidative stress and myosin light-chain phosphorylation, which results in vasoconstriction, depolarization due to increased calcium sensitization, increased peripheral resistance, increased expression of endothelin-1 and the potentiated effects of beta-adrenoreceptors leading to hypertension [6]. *C. sativum* and CL are known antioxidants and blood pressure-lowering agents. Invasive blood pressure measurement after administering CS and CL in the normotensive anesthetized rats demonstrated a significant fall in blood pressure at their highest tested dose. *C. sativum* and CL administration from the eighth week along with arsenic decreased the oxidative stress and lowered the blood pressure in treated animals, evidenced from the peroxidation assay and alleviated blood pressure monitored through the non-invasive blood measurement technique. The flavonoid content in CS and CL also favors anti-oxidant, anti-inflammatory and anti-allergic effects. High Performance Liquid Chromatography analysis of the CS and CL extracts showed various flavonoids. The presence of these flavonids was also in accordance with earlier findings that described their anti-inflammatory, antioxidant and diuretic properties [29].

In order to elucidate insights into mechanism, CS and CL were subjected for their effects on isolated rat aortic tissue. Both CS and CL showed complete relaxation in response to high K^+^ and P.E-induced contraction. This showed that the relaxant effect of the test substances might be due to the α antagonist/Ca^++^ antagonist affect. It is known that any substance with the ability to relax high K^+^-induced contraction might indicate its interference with voltage-dependent calcium channels, which results in relaxation [30]. In this experiment, the administration of arsenic caused an increase in the levels of the serum electrolytes Na^+^ and K^+^. Arsenic affects biological membranes through peroxidation of unsaturated fatty acids. This peroxidation disrupts membrane fluidity and integrity, which leads to delocalization of Na^+^-K^+^ ATPase resulting in electrolyte imbalance. Increased sodium levels lead to hypertension by increased reabsorption and electrolyte retention, retaining sodium passed through renal tubules and stimulating the renin–angiotensin–aldosterone system in the brain. The stimulation of this system may cause increased blood pressure via angiotensin II and increased aldosterone-mediated oxidative stress by stimulating the sympathetic nervous system (beta adrenoreceptor stimulation). Raised levels of potassium lead to hyperkalaemia, which results in disrupted heart rhythms, vasoconstriction and thus, hypertension [31]. Treatment with CS and CL showed a decrease in Na^+^ and K^+^ levels that is in line with previous studies [12,32]. The decrease in serum electrolytes may be due to the antioxidant potential and ROS scavenging activity resulting in reduced oxidative stress. *C. sativum* is also capable of inhibiting beta adrenoreceptor-stimulated ROS generation. Similarly, CL is capable of influencing vascular permeability and enhancing capillary resistance favoring the results of the study [33].

A significant reduction in nitric oxide levels was observed in the sodium arsenate-treated rats. NO is a vasodilator that aids in modulating vascular tone, blood pressure and hemodynamics [34]. Arsenic disrupts the endothelial system by causing oxidative stress, which forms iNOS instead of eNOS, thus reducing eNOS activity and BH_4_ levels (important co-factors in eNOS coupling), increasing NADPH oxidase activity and decreasing antioxidants. When the CS and CL were administered at defined doses in arsenic-fed rats, serum NO levels were restored, which favors attenuation of oxidative stress, ultimately resulting in curing hypertension [35,36]. The integrity of endothelium was carried out by observing Ach-induced relaxation on the aorta. Plant-treated groups showed a reversal of endothelial functioning, which was evident with Ach-treated vasorelaxation in the treated groups versus the diseased group.

Arsenic administration caused a significant change in the lipid profile. Arsenic elevated levels of cholesterol, VLDL, LDL and triglycerides and decreased HDL. The lipid profile is an indication of atherosclerosis, a major risk factor for hypertension and cardiovascular diseases. This change in lipid profile was due to elevated plasma-free fatty acids and the inhibition of the reverse cholesterol transport. Furthermore, arsenic affects lipid homeostasis and oxidative stress, which mediates inflammation, ultimately leading to endothelial dysfunction and an increase in hypertension due to impaired nitric oxide homeostasis. [37]. When arsenic-fed rats were treated with CS and CL, there was a marked reduction in cholesterol and increase in the HDL levels of the rats. *C. sativum* reduced the cholesterol levels and its accumulation along arteries and veins by inhibiting the HMG-CoA reductase enzyme. The breakdown of cholesterol into neutral sterols and faecal bile acids enhanced the activity of the lecithin-cholesterol acyl transferase (LCAT) enzyme [35]. *C. limon* also helps in decreasing levels of cholesterol, triglycerides and other lipid markers by inhibiting the HMG-CoA reductase enzyme and up-regulation of LDL receptors. Both test drugs showed positive results in treating hyperlipidemia, which also leads to hypertension and other CVDs.

Arsenic causes oxidative stress, which disrupts liver function by disrupting the cell structure through lipid peroxidation. This is due to the activation of some enzymes such as CYP 450, JNK, p38 and MAPK that are responsible for apoptosis resulting in hepatotoxicity. The decrease in antioxidant enzymes also leads to hepatotoxicity [38]. In our study, due to arsenic administration in rats for twelve weeks, the liver function levels of AST, ALT and bilirubin were increased compared to naive rats. The administration of arsenic for a long time leads to fatty liver and fibrosis due to an increase in serums ALT and AST. Increased ALT levels relate to hypertension indirectly, yet there is no clear mechanism defined. Possibly, it may be due to the increased responses of TNF- α and interleukin [32]. Due to arsenic exposure, Na^+^/K^+^ ATPase activity was decreased, and reduced Na^+^/K^+^ ATPase activity leads to an electrolyte imbalance that causes hypertension [39]. When CS was administered at defined doses in arsenic-fed rats, it showed a significant response in normalizing levels of ALT, AST, ALP and other liver markers. It restores cell structures by decreasing lipid peroxidation and tissue degeneration [40]. Our findings are in agreement with [40], as in our study CS also ameliorates liver markers. When *C. limon* was administered at defined dose in arsenic-fed rats, it also decreased the AST, ALT and cholesterol levels and these results are in agreement with [41]. Treating oxidative stress normalized the high levels of liver enzymes, thus resulting in a hepatoprotective effect. Chronic arsenic administration in this experiment caused nephrotoxicity by producing oxidative stress, decreasing antioxidant enzymes and increasing kidney MDA levels, which are also an indication of oxidative damage [42]. In this study, arsenic-fed rats showed high serum creatinine and urea levels. When *C. sativum* and *C. limon* were administered in arsenic-fed rats, there was a significant decrease in serum creatinine, urea and MDA levels. The results showed reduced levels of ceatinine, urea, MDA and oxidative stress. *C. limon* peel was effective in decreasing levels of serum creatinine, urea, AST and ALT by reducing oxidative stress according to previous studies [8]. Our study demonstrated that CS and CL have an anti-oxidant potential to reduce the progress of kidney damage caused by arsenic.

## 5. Conclusions

This study establishes that *C. sativum* and *C. limon* treatments mitigate arsenic-induced hypertension and arsenic-mediated oxidative stress, possibly through their endothelium-protective, and anti-oxidant activities. These plants also showed nephroprotective and hepatoprotective effects by decreasing lipid peroxidation as observed in the MDA assay. The anti-oxidant effects were depicted by DPPH assay and restored NO levels. The presence of phytoconstitutes had an added effect in lowering blood pressure. Thus, this study provides a rationale for the medicinal use of CS and CL in hypertension and also against arsenic-induced cardiovascular complications. Further studies to standardize the extracts against reported phytoconstituents and molecular studies are suggested.

## Figures and Tables

**Table 1:** Effect of *C. sativum, C. limon* and their combinations on serum lipid profile.

Groups	TC (mg/dL)	TG (mg/dL)	HDL (mg/dL)	LDL (mg/dL)	VLDL (mg/dL)
Normal	84.20 ± 0.583	62.20 ± 0.8602	28.00 ± 0.9487	25.40 ± 0.50	11.00 ± 1.304
Diseased	100.8 ^###^ ± 1.7	127.8 ^###^ ± 6.89	18.40 ^###^ ± 0.50	67.20 ^###^ ± 2.41	24.80 ^###^ ± 1.71
Ar + positive control	92.20 ^***^ ± 0.58	103.2 ^***^ ± 5.26	23.60 ^***^ ± 0.50	58.80 ^*^ ± 1.80	16.20 ^***^ ± 0.86
Ar + CS high dose	76.00 ^***^ ± 0.70	82.20 ^***^ ± 2.08	26.20 ^***^ ± 0.58	46.80 ^***^ ± 2.26	15.80 ^***^ ± 0.58
Ar + CL high dose	90.00 ^***^ ± 1.14	80.80 ^***^ ± 1.93	21.80 ^***^ ± 0.37	52.60 ^***^ ± 1.60	15.60 ^***^ ± 1.03
Ar + CL + CS low doses	86.60 ^***^ ± 1.96	90.00 ^***^ ± 2.302	20.80 ^**^ ± 0.37	55.20 ^***^ ± 1.93	15.80 ^***^ ± 0.58
Ar + CL + CS high doses	67.60 ^***^ ± 0.92	72.80 ^***^ ± 2.65	23.40 ^***^ ± 0.50	39.00 ^***^ ± 1.30	13.40 ^***^ ± 1.03

Where normal: normal control. Diseased: arsenic fed hypertensive control. Ar + CS: arsenic with *C. sativum* (500 mg/kg). Ar + CL: arsenic with *C. limon* (100 mg/kg). Ar + CL + CS (low doses): arsenic with low doses of *C. limon and C. sativum* (50 + 250 mg/kg). Ar + CL + CS (high doses): arsenic with high doses of *C. limon and C. sativum* (70 + 350 mg/kg). Data are represented as mean ± SEM. *n* = 5. ^###^ *p* < 0.001 shows comparison between normal and diseased group by using Student *t* test. ^***^ *p* < 0.001, ^**^ *p* < 0.01 and ^*^ *p* < 0.05 indicate comparison between diseased and treatment groups by using one-way ANOVA analysis of variance.

## Data Availability

Data are available from authors upon suitable request.

## Supplementary Materials

The following are available online at https://www.mdpi.com/article/10.3390/life12111842/s1, Figure S1. H and E staining of heart tissue of normal (A), diseased (B), Positive control (C), *C. sativum* high dose (D), *C. limon* high dose (E), low dose combination (F) and high dose combination (G). Figure S2. H and E staining of aorta tissue of normal (A), diseased (B), Positive control (C), *C. sativum* high dose (D), *C. limon* high dose (E), low dose combination (F) and high dose combination (G). Figure S3. H and E staining of kidney tissue of normal (A), diseased (B), Positive control (C), *C. sativum* high dose (D), *C. limon* high dose (E), low dose combination (F) and high dose combination (G). Figure S4. H and E staining of liver tissue of normal (A), diseased (B), Positive control (C), *C. sativum* high dose (D), *C. limon* high dose (E), low dose combination (F) and high dose combination (G).
