# Supplementary material for: Evaluation of the Cardiovascular Effects of Coriandrum sativum and Citrus limon to Treat Arsenic-Induced Endothelial Damage and Hypertension in Rats
Source: Life (Basel). 2022 Nov 10;12(11):1842. doi: 10.3390/life12111842 (PMC9695286; doi:10.3390/life12111842)
Supplement: Supplementary file 1 [file life-12-01842-s001.zip › life-1949353-supplementary.pdf]

life

MDPI

Supplementary Figures

Histopathological examination of heart tissue:

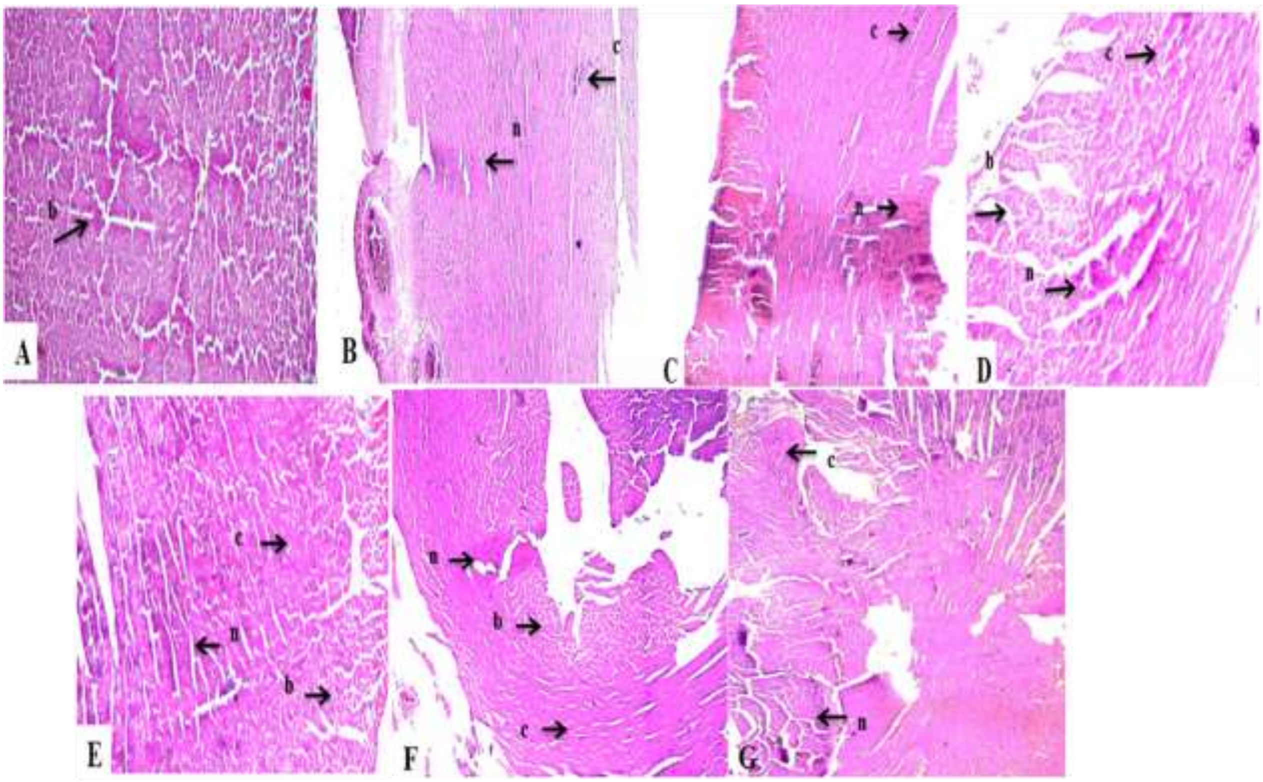

Figure S1: H & E staining of heart tissue of normal (A), diseased (B), Positive control (C), *C. sativum* high dose (D), *C. limon* high dose (E), low dose combination (F) and high dose combination (G).

Histopathological examination of aorta tissue:

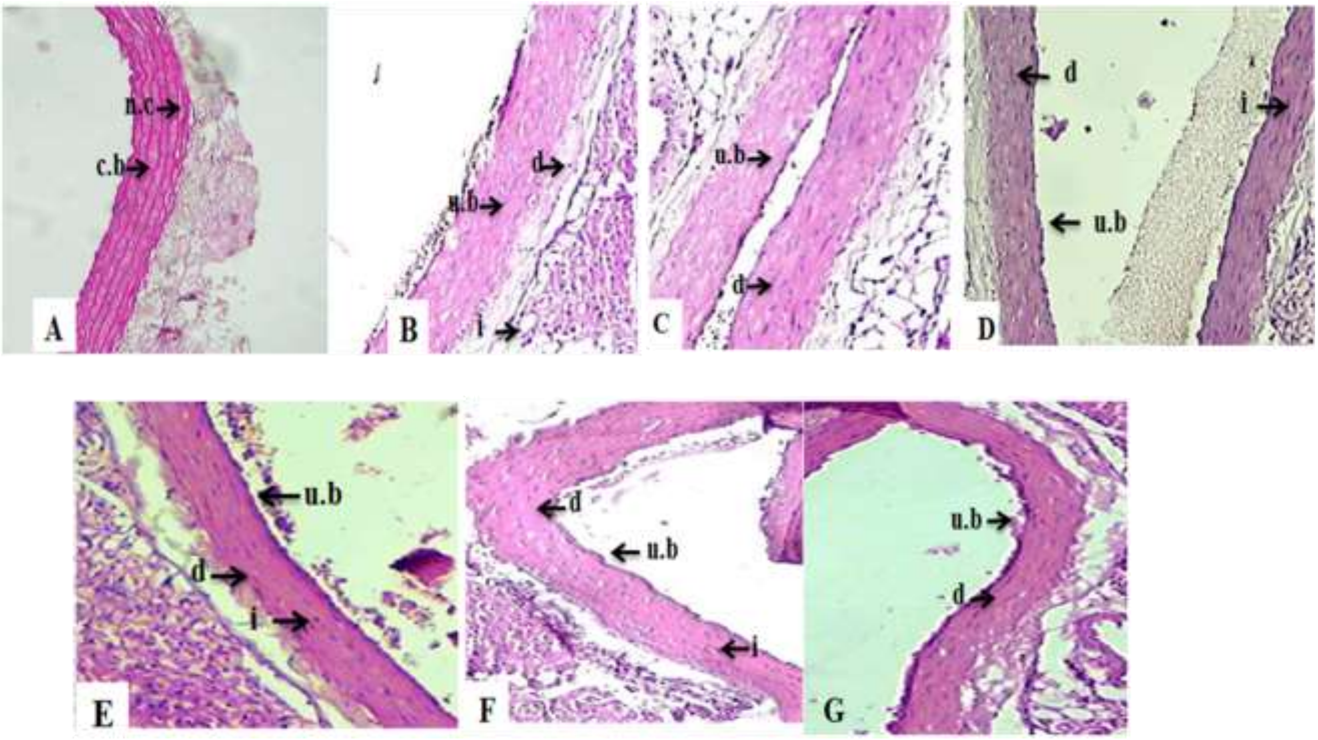

Figure S2: H & E staining of aorta tissue tissue of normal (A), diseased (B), Positive control (C), *C. sativum* high dose (D), *C. limon* high dose (E), low dose combination (F) and high dose combination (G)

Histopathological examination of kidney tissue:

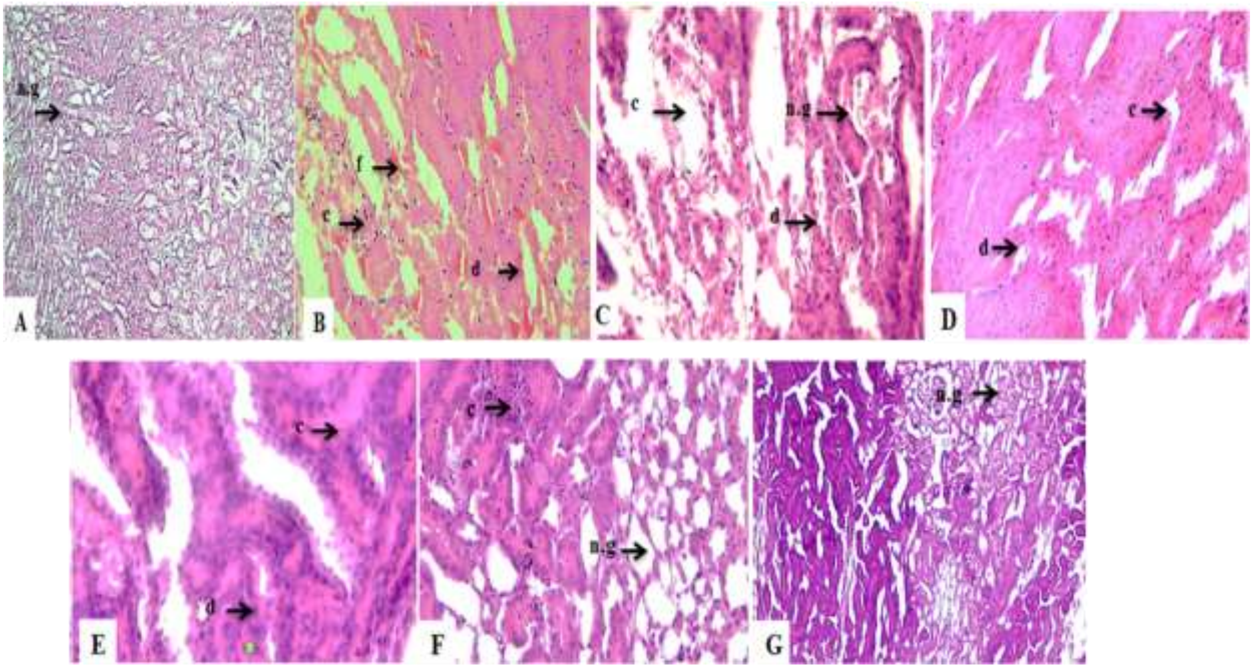

Figure S3: H & E staining of kidney tissue of normal (A), diseased (B), Positive control (C), *C. sativum* high dose (D), *C. limon* high dose (E), low dose combination (F) and high dose combination (G)

Histopathological examination of liver tissue

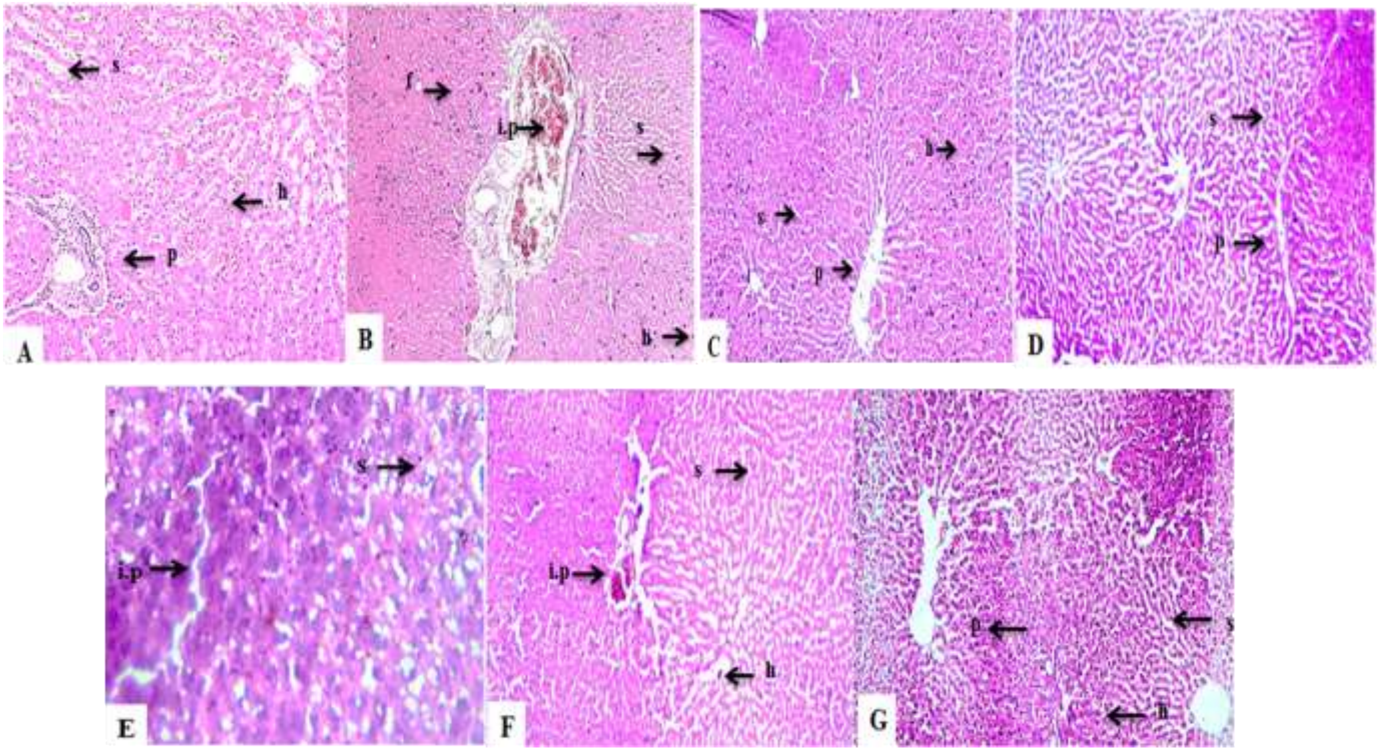

Figure S4: H & E staining of liver tissue of normal (A), diseased (B), Positive control (C), *C. sativum* high dose (D), *C. limon* high dose (E), low dose combination (F) and high dose combination (G)
